# Contributions of upper gut hormones and motility to the energy intake‐suppressant effects of intraduodenal nutrients in healthy, lean men – a pooled‐data analysis

**DOI:** 10.14814/phy2.12943

**Published:** 2016-09-13

**Authors:** Gudrun Schober, Kylie Lange, Robert E. Steinert, Amy T. Hutchison, Natalie D. Luscombe‐Marsh, Maria F. Landrock, Michael Horowitz, Radhika V. Seimon, Christine Feinle‐Bisset

**Affiliations:** ^1^ University of Adelaide Discipline of Medicine Adelaide Australia; ^2^ NHMRC Centre of Excellence in Translating Nutritional Science to Good Health University of Adelaide Adelaide Australia; ^3^ South Australian Health and Medical Research Institute Adelaide Australia; ^4^ CSIRO Animal, Food and Health Sciences Adelaide Australia; ^5^ Boden Institute of Obesity, Nutrition, Exercise & Eating Disorders, University of Sydney Sydney Australia

**Keywords:** Appetite perceptions, determinants of energy intake, cholecystokinin, glucagon‐like peptide‐1, peptide YY, pyloric pressures

## Abstract

We have previously identified pyloric pressures and plasma cholecystokinin (CCK) concentrations as independent determinants of energy intake following administration of intraduodenal lipid and intravenous CCK. We evaluated in healthy men whether these parameters also determine energy intake in response to intraduodenal protein, and whether, across the nutrients, any predominant gastrointestinal (GI) factors exist, or many factors make small contributions. Data from nine published studies, in which antropyloroduodenal pressures, GI hormones, and GI /appetite perceptions were measured during intraduodenal lipid or protein infusions, were pooled. In all studies energy intake was quantified immediately after the infusions. Specific variables for inclusion in a mixed‐effects multivariable model for determination of independent predictors of energy intake were chosen following assessment for collinearity, and within‐subject correlations between energy intake and these variables were determined using bivariate analyses adjusted for repeated measures. In models based on all studies, or lipid studies, there were significant effects for amplitude of antral pressure waves, premeal glucagon‐like peptide‐1 (GLP‐1) and time‐to‐peak GLP‐1 concentrations, GLP‐1 AUC and bloating scores (*P* < 0.05), and trends for basal pyloric pressure (BPP), amplitude of duodenal pressure waves, peak CCK concentrations, and hunger and nausea scores (0.05 < *P* ≤ 0.094), to be independent determinants of subsequent energy intake. In the model including the protein studies, only BPP was identified as an independent determinant of energy intake (*P* < 0.05). No single parameter was identified across all models, and effects of the variables identified were relatively small. Taken together, while GI mechanisms contribute to the regulation of acute energy intake by lipid and protein, their contribution to the latter is much less. Moreover, the effects are likely to reflect small, cumulative contributions from a range of interrelated factors.

## Introduction

In healthy humans, energy intake and expenditure are, in most cases, balanced precisely over long periods of time, so that body weight is stable. This energy homeostasis is controlled by complex interactions between central and peripheral feedback signals, including neurohumoral responses to ingested food (Woods et al. 2000; Cummings and Overduin 2007). The arrival of nutrients in the small intestine modulates a number of gastrointestinal (GI) functions, including gastroduodenal motility (Heddle et al. 1988b; Cook et al. 1997; Pilichiewicz et al. 2007b), associated with a slowing of gastric emptying (Heddle et al. 1989), and the stimulation of putative eating‐inhibitory gut hormones, including cholecystokinin (CCK) (Feltrin et al. 2007; Pilichiewicz et al. 2007b), glucagon‐like peptide‐1 (GLP‐1) (Feinle et al. 2002; Pilichiewicz et al. 2007b; Ryan et al. 2013), and peptide YY (PYY) (Feltrin et al. 2007; Pilichiewicz et al. 2007b), as well as the suppression of ghrelin (Parker et al. 2005; Feltrin et al. 2006; Cukier et al. 2008), the only known orexigenic gut hormone (Tschop et al. 2000; Cummings et al. 2001; Wren et al. 2001). The stimulation of phasic and tonic pyloric pressures is critical for the slowing of gastric emptying by nutrients (Heddle et al. 1989). Among the macronutrients, lipid appears to have the most potent effects to modulate GI motor and hormone functions (Andrews et al. 1998; Pilichiewicz et al. 2007a,b; Seimon et al. 2009a; Ryan et al. 2013). Our pooled data analysis of eight studies from our laboratory, in which antropyloroduodenal pressures, GI hormones, and GI/appetite perceptions were measured during intraduodenal lipid, or intravenous CCK, infusions, indicated that the magnitude of the stimulation of pyloric pressures and plasma CCK concentrations is independent determinants of subsequent energy intake in healthy men (Seimon et al. 2010), consistent with the concept that both pyloric pressures and CCK are important, in the acute regulation of energy intake.

Protein is generally regarded as the most satiating macronutrient (Latner and Schwartz 1999; Weigle et al. 2005; Batterham et al. 2006; Westerterp‐Plantenga et al. 2009), although recent studies from our laboratory in lean subjects have shown that orally ingested high‐protein or high‐fat meals (Brennan et al. 2012), and intraduodenal infusions of pure fat or protein (Ryan et al. 2013), have comparable effects to reduce energy intake. However, in these studies, despite equipotent effects on energy intake, the effects of intraduodenal protein to stimulate pyloric pressures and plasma CCK and GLP‐1 concentrations were much less than those of intraduodenal lipid, whereas protein had more potent effects to stimulate plasma insulin and glucagon (Ryan et al. 2013). These observations, accordingly, suggest that the energy intake‐suppressant effects of protein may not be mediated by these GI mechanisms to the same extent as for lipid, which warrants further evaluation. Over the last years, we have performed a number of studies relating to the effects of protein and amino acids on GI function and energy intake (Ryan et al. 2012, 2013; Steinert et al. 2014, 2015). This analysis was stimulated by the recognition that, in our previous pooled analysis (Seimon et al. 2010), (1) effects of protein were not evaluated; and (2) we included studies utilizing intravenous hormone infusions, particularly exogenous CCK, which is known to potently stimulate pyloric pressures and increase plasma CCK concentrations to supraphysiological levels, and, thus, may have led to an overestimation of the role of these two outcomes. Because of the potent energy intake‐suppressant, but more modest GI, effects of protein, we hypothesized, based on our recent study (Ryan et al. 2013), that, in contrast to lipid, GI mechanisms would not be identified as independent determinants of energy intake in the varying protein interventions/loads used.

## Methods

### Subjects

A total of 117 healthy, normal‐weight men, aged 26 ± 3 years, and with a BMI of 22.6 ± 0.8 kg/m^2^, were included in this pooled data analysis (Feltrin et al. 2004, 2006, 2008; Little et al. 2005; Pilichiewicz et al. 2007b; Seimon et al. 2009a; Ryan et al. 2012, 2013; Steinert et al. 2014, 2015) **(**Table 1 **)**. All participants were unrestrained eaters with a score of ≤12 on the eating‐restraint component of the three‐factor eating questionnaire (Stunkard and Messick 1985). Subjects who smoked, consumed >20 g of alcohol/day, had a history of GI symptoms, or took medication known to affect appetite, eating, or GI function were excluded. Each subject provided informed, written consent before their inclusion, and the Royal Adelaide Research Ethics Committee approved the study protocols, and studies were performed in accordance with the Declaration of Helsinki.

**Table 1:** Subject and protocol details for each study included in the data analysis

Study no.	Authors (References)	Subject (*n*)	Age (year)	BMI (kg/m^2^)	Study intervention
1	Feltrin et al. (2004, 2006)^a^	7^b^	24 ± 4	22.0 ± 1.6	ID saline (control), 0.375 kcal/min decanoic or dodecanoic acid for 90 min. Buffet meal at 90 min.
2	Little et al. (2005)	13	23 ± 2	23.6 ± 0.5	ID saline, 0.1, 0.2, or 0.4 kcal/min dodecanoic acid for 90 min. Buffet meal at 90 min.
3	Pilichiewicz et al. (2007b)	16	31 ± 3	23.8 ± 0.5	ID saline, 0.25, 1.5, or 4 kcal/min of 10% Intralipid for 50 min. Buffet meal at 50 min.
4	Feltrin et al. (2008)	13	26 ± 2	22.9 ± 0.6	ID saline, 0.4 kcal/min of dodecanoic, or oleic acid for 60 min. Buffet meal at 60 min.
5	Seimon et al. (2009b)	10	25 ± 3	22.8 ± 0.4	ID saline, 2.8 kcal/min of fat emulsions with droplet sizes of 0.26, 30, or 170 μm for 120 min. Buffet meal at 120 min.
6	Ryan et al. (2012)	16	27 ± 3	22.1 ± 0.6	ID saline, 0.5, 1.5, or 3 kcal/min of 18.1% hydrolyzed whey protein for 60 min. Buffet meal at 60 min.
7	Ryan et al. (2013)	20	27 ± 3	22.4 ± 0.4	ID saline, 3 kcal/min of 18.1% hydrolyzed whey protein or 20% Intralipid for 90 min. Buffet meal at 90 min.
8	Steinert et al. (2014)	10	27 ± 9	22.5 ± 2.1	ID saline, 0.075 or 0.15 kcal/min of l‐tryptophan for 90 min. Buffet meal at 90 min.
9	Steinert et al. (2015)	12	25 ± 2	21.9 ± 0.4	ID saline, 0.15 or 0.45 kcal/min of l‐leucine for 90 min. Buffet meal at 90 min.

ID, intraduodenal. Data are means ± SEM.

a Parts of the hormone data were analyzed and published separately, which resulted in two publications.

b Plasma was available in only seven of the eight subjects included in the analyses.

### Study design

Data from nine published studies (Feltrin et al. 2004, 2006, 2008; Little et al. 2005; Pilichiewicz et al. 2007b; Seimon et al. 2009a; Ryan et al. 2012, 2013; Steinert et al. 2014, 2015), performed in our laboratory between 2003–13, were pooled for this analysis. All studies used identical methods and techniques and evaluated the outcome measures of interest **(**Table 2 **).** While, as in our previous pooled analysis (Seimon et al. 2010), data were analyzed using the same statistical tests that would be appropriate for an individual participant meta‐analysis, it would be inappropriate to refer to our analysis as such because the studies included were not identified through a systematic review (Simmonds et al. 2005).

**Table 2:** Variables measured in each study

Study no.	Authors (References)	APD pressures				Hormones						Perceptions					
		APWs	IPPWs	BPP	DPWs	Ghr	CCK	PYY	GLP‐1	Insulin	Glu^b^	Hunger	Desire to eat	Prosp. cons.	Fullness	Nausea	Bloating
1	Feltrin et al. (2007)	X	X	X	X		X		X			X	X	X	X	X	X
	Feltrin et al. (2004)^a^					X		X									
2	Little et al. (2005)	X	X	X	X		X		X			X	X	X	X	X	X
3	Pilichiewicz et al. (2007b)	X	X	X	X		X	X				X	X	X	X	X	X
4	Feltrin et al. (2008)	X	X	X	X		X	X				X	X	X	X	X	X
5	Seimon et al. (2009b)	X	X	X	X		X	X				X	X	X	X	X	X
6	Ryan et al. (2012)	X	X	X	X	X	X	X	X	X		X	X	X	X	X	X
7	Ryan et al. (2013)	X	X	X	X	X	X	X	X	X	X	X	X	X	X	X	X
8	Steinert et al. (2013)	X	X	X	X	X	X	X	X	X	X	X	X	X	X	X	X
9	Steinert et al. (2016)	X	X	X	X	X	X	X	X	X	X	X	X	X	X	X	X

APD, antropyloroduodenal; APWs, antral pressure waves; IPPWs, isolated pyloric pressure waves; BPP, basal pyloric pressure; DPWs, duodenal pressure waves; Ghr, Ghrelin; CCK, cholecystokinin; GLP‐1, glucagon‐like peptide 1; PYY, peptide YY; Glu, Glucagon; Prosp. cons., prospective consumption; X, variable measured.

a Parts of the hormone data were analyzed and published separately, which resulted in two publications.

b Not included in the subsequent statistical analyses because glucagon was only evaluated in a small number of studies.

### Study protocols

Each study assessed the effects of ID infusions of nutrients (e.g., triglyceride or fatty acids, protein or amino acids) and appropriate control solutions on antropyloroduodenal pressures, GI hormone release, GI/appetite perceptions, and energy intake. Information regarding the study interventions in each study is provided in Table 1 **.** Detailed study protocols have been described in the original publications (Feltrin et al. 2004, 2006, 2008; Little et al. 2005; Pilichiewicz et al. 2007b; Seimon et al. 2009a; Ryan et al. 2012, 2013; Steinert et al. 2014, 2015). Briefly, in all studies, participants attended the laboratory in the Discipline of Medicine, Royal Adelaide Hospital, at 0830 h after an overnight fast, for multiple study visits in randomized order. On arrival, a small‐diameter manometric catheter (outer diameter: 3.5 mm; total length: 100 cm; Dentsleeve International Ltd, Mississauga, Ontario, Canada) was inserted through an anesthetized nostril into the stomach and allowed to pass into the duodenum by peristalsis (Heddle et al. 1989). The catheter consisted of 16 side holes, spaced at 1.5 cm intervals, to measure pressures in the antrum (channels 1–6), pylorus (channels 7–9; 4.5‐cm sleeve sensor), and duodenum (channels 10–16), and an additional side hole located in the duodenum for ID infusions. An intravenous cannula was placed into a right forearm vein for regular blood sampling for subsequent measurements of plasma GI hormone, and/or insulin, concentrations. Once the catheter was placed correctly, as described (Heddle et al. 1988b), fasting motility was observed until the occurrence of phase III of the interdigestive migrating motor complex. Immediately after the end of phase III activity, in phase I, a baseline blood sample was taken, and the subject completed a visual analog scale (VAS) questionnaire to assess GI perceptions (Parker et al. 2004). ID infusion of nutrient or control solutions commenced, and antropyloroduodenal pressures were recorded throughout the infusion period. Further blood samples were collected, and VAS questionnaires completed, at regular intervals. At the end of each ID infusion, the catheter and cannula were removed, and the subject was presented with a standardized, cold, buffet‐style meal (Nair et al. 2009). The meal consisted of white and whole‐meal breads, sliced, cold meats (ham, chicken), cheese, lettuce, tomato, cucumber, mayonnaise, butter, apple, banana, yoghurt, chocolate custard, fruit salad, iced coffee, orange juice, and water. Subjects were allowed to eat as much as they wished for up to 30 min, until they felt comfortably full.

### Measurements

#### APD pressures

APD pressures were digitized using a computer‐based system running commercially available software and analyzed for (1) the number and amplitude of antral and duodenal pressure waves; (2) basal pyloric pressure (BPP; “tone”); and (3) the number and amplitude of isolated pyloric pressure waves (IPPWs) (Heddle et al. 1988b). Antral and duodenal pressure waves were expressed as total numbers and mean amplitudes (mmHg). For IPPWs, number of premeal IPPWs (i.e., during the 15‐min period immediately before the buffet meal), the peak number, and the time (min)‐to‐peak number during ID infusions, total number, and areas under the curve (AUCs, calculated using the trapezoidal rule) of total number (min) and amplitude (mmHg·min) were quantified. BPP was expressed as peak pressures (mmHg), time (min)‐to‐peak pressure, and AUC (mmHg·min) over the infusion period.

#### Hormone concentrations

Venous blood samples were collected, processed, and stored for later analysis of total ghrelin, CCK, total PYY, total GLP‐1, and insulin. Plasma total ghrelin (pmol/L) was measured by radioimmunoassay, without peptide extraction (Phoenix Pharmaceuticals, Mountain View, CA). No cross‐reactivities with any relevant molecule have been reported. Intra‐ and interassay coefficients of variation (CVs) were 5.0% and 15.0%, respectively. The detection limit was 13 pmol/L. Plasma CCK‐8 (pmol/L) was measured by radioimmunoassay after ethanol extraction using an adaptation of a previously published method (Santangelo et al. 1998). Intra‐ and interassay CVs were 8.3% and 12.6%, respectively. The detection limit was 1 pmol/L. Plasma total GLP‐1 (pmol/L) was measured by radioimmunoassay (Millipore, Billerica, MA). The antibody used did not cross‐react with glucagon, gastric inhibitory polypeptide, or other gut or pancreatic peptides. Intra‐ and interassay CVs were 4.8% and 6.8%, respectively. The detection limit was 3 pmol/L. Plasma total PYY (pmol/L) was measured by radioimmunoassay (Linco Research, St. Charles, MO). Intra‐ and interassay CVs were 5.3% and 7.0%, respectively. The detection limit was 2.5 pmol/L. Plasma insulin (mU/L) was measured by an ELISA assay (Mercodia, Uppsala, Sweden). Intra‐ and interassay CVs were 2.9% and 8.8%, respectively. The detection limit was 1 mU/L.

For CCK, premeal and peak concentrations (pmol/L), the time (min)‐to‐peak concentrations, and the AUC (pmol·min/L) over the infusion period were calculated. For ghrelin, PYY, GLP‐1, and insulin, premeal concentrations (pmol/L or mU/L, as appropriate) and AUC (pmol·min/L or mU·min/L, as appropriate) were calculated. Peak concentrations were not calculated for ghrelin, PYY, GLP‐1, and insulin because concentrations were shown to continue to rise, or decline (for ghrelin), throughout the infusion period.

#### GI/appetite perceptions and energy intake

Hunger, desire to eat, prospective consumption, fullness, nausea, and bloating were rated using validated 100‐mm VAS scales (Parker et al. 2004) and expressed as AUCs over the infusion period (mm·min). Energy intake (kJ) was determined from the food consumed (g) at the buffet meal, by weighing the different food items before and after consumption, and then calculating their energy content using commercially available software (Foodworks; Xyris Software, Highgate Hill, QLD, Australia) (Feltrin et al. 2004; Ryan et al. 2013).

### Statistical analysis

Statistical analyses were performed using SPSS 22 software (2013, IBM Corp, Armonk, NY).

Within‐subject correlations, adjusted for repeated measures, were performed to assess the strength of bivariate relations between energy intake and each hormone, motility, and GI/appetite perception variable (Bland and Altman 1995). Variables were then entered simultaneously into a multivariable maximum‐likelihood linear mixed‐effects model, adjusted for repeated visits per subjects and the clustering of subjects within studies, to determine the independent effects of each variable on energy intake (Raudenbush and Bryk 2002). This is equivalent to the one‐step analysis approach in a meta‐analysis of individual participant data (Riley et al. 2010).

Analyses were conducted for all studies combined (models 1a, 1b), for the lipid‐based studies (models 2a, 2b), and for the protein‐based studies (model 3). Because GLP‐1 and PYY were not measured in all studies, the multivariable analyses were conducted as two separate models for ‘all studies’ and for the ‘lipid studies’. Models 1a and 2a considered all variables measured across all studies plus PYY. Models 1b and 2b considered all variables measured across all studies plus GLP‐1. Variables were included in the multivariable models after screening for multicollinearity and excluding related parameters from the same underlying motility, hormone, or GI/appetite perception variable, as indicated by the variance inflation factors and condition indices. Where related variables were collinear, parameters were selected for inclusion based on how they best reflected the observed physiological response over the study periods. These selections were made without referring to the model results to avoid post hoc variable selection. Further exclusion of variables was conducted for the lipid and protein subgroups, to reduce the models to a number of parameters appropriate for the reduced sample sizes. For this purpose, F‐statistics were used to exclude the variables that least contributed to the model, analogous to backward variable selection. Thus, the following variables were excluded in the process: IPPW time‐to‐peak number, IPPW number AUC, BPP time‐to‐peak pressure, CCK premeal, and CKK time to peak. Based on the same principles, further variables were excluded to reach a final model with an appropriate number of parameters, including, from the lipid subgroup, mean amplitude of antral pressure waves, IPPW amplitude AUC, BPP AUC, mean amplitude of duodenal pressure waves, AUCs for CCK, PYY, GLP‐1, prospective consumption and fullness, and from the protein subgroup, additionally IPPW peak number, number of duodenal pressure waves, and desire to eat AUC. At each step, the models were examined for consistency and robustness of the parameter estimates.

Data are expressed as means ± standard error of the mean (SEM) or standard deviation (SD), as indicated, and differences were considered significant when *P *<* *0.05.

## Results

### Bivariate correlation analyses

Within‐subject correlations between energy intake and each of the measured variables are presented in Table 3 **.** Collinearity was present between a number of variables; thus, related variables from the same underlying motility, hormone, or GI/appetite perception variable were excluded from the multivariate model to guarantee a robust estimation of the regression effects.

**Table 3:** Within‐subject correlations between energy intake and GI motor, hormone, and GI/appetite perception variables

Variable	All studies				Lipid studies				Protein studies			
	*n* a	Mean ± SD	*r*	*P*	*n* a	Mean ± SD	*r*	*P*	*n* a	Mean ± SD	*r*	*P*
Antral pressure waves												
Number, total	400	59 ± 86	0.14	0.019	256	57 ± 98	0.13	0.086	164	77 ± 80	0.21	0.028
Amplitude, mean (mmHg)	400	62 ± 80	0.23	<0.001	256	52 ± 87	0.22	0.004	164	104 ± 105	0.26	0.006
Isolated pyloric pressure waves												
Number premeal/15 min	384	9 ± 12	−0.17	0.004	240	9 ± 12	−0.09	0.229	164	8 ± 11	−0.32	0.001
Peak number/15 min	384	20 ± 14	−0.24	<0.001	240	21 ± 14	−0.22	0.004	164	17 ± 12	−0.34	<0.001
Time‐to‐peak number (min)	359	29 ± 22	0.05	0.390	216	28 ± 22	0.18	0.031	163	31 ± 23	−0.16	0.104
Total number	400	53 ± 60	−0.19	<0.001	256	56 ± 67	−0.14	0.066	164	45 ± 43	−0.38	<0.001
AUC number (min)	384	727 ± 784	−0.21	<0.001	240	815 ± 886	−0.18	0.025	164	540 ± 530	−0.38	<0.001
AUC amplitude (mmHg·min)	384	1699 ± 1105	−0.10	0.109	240	1863 ± 1126	−0.01	0.902	164	1404 ± 1021	−0.24	0.010
Basal pyloric pressures												
Peak pressure (mmHg)	393	4.0 ± 5.0	−0.14	0.023	249	4.4 ± 5.4	−0.13	0.094	164	3.0 ± 4.1	−0.28	0.003
Time‐to‐peak pressure (min)	387	31 ± 24	0.03	0.611	243	29 ± 24	0.08	0.298	164	37 ± 25	0.00	0.971
AUC (mmHg·min)	393	62 ± 206	−0.11	0.077	249	77 ± 232	−0.12	0.109	164	38 ± 148	−0.16	0.088
Duodenal pressure waves												
Number, total	397	450 ± 336	0.21	<0.001	253	451 ± 376	0.28	<0.001	164	480 ± 270	0.14	0.153
Amplitude, mean (mmHg)	400	56 ± 47	0.11	0.063	256	44 ± 43	0.14	0.068	164	87 ± 53	0.12	0.213
Ghrelin												0.148
Premeal (pmol/L)	181	266 ± 213	0.34	<0.001	47	199 ± 155	0.62	0.001	147	275 ± 221	0.25	0.012
AUC (pmol·min/L)	181	18,866 ± 13,491	0.34	<0.001	47	19,034 ± 13,164	0.59	0.001	147	18,120 ± 13,242	0.22	0.028
Cholecystokinin												
Premeal (pmol/L)	382	4.6 ± 3.4	−0.35	<0.001	232	5.1 ± 3.9	−0.37	<0.001	170	3.6 ± 2.1	−0.45	<0.001
Peak concentration (pmol/L)	382	6.0 ± 4.5	−0.25	<0.001	232	6.9 ± 5.3	−0.26	0.001	170	4.4 ± 2.3	−0.43	<0.001
Time‐to‐peak conc (min)	352	29 ± 25	−0.19	0.003	202	27 ± 25	−0.18	0.042	168	32 ± 24	−0.24	0.012
AUC (pmol·min/L)	359	338 ± 265	−0.24	<0.001	209	392 ± 325	−0.27	0.001	170	254 ± 111	−0.45	<0.001
Peptide YY												
Premeal (pmol/L)	309	42.9 ± 27.1	−0.25	<0.001	166	47.7 ± 33.6	−0.30	0.001	156	36.4 ± 14.8	−0.30	0.002
AUC (pmol·min/L)	309	5260 ± 7830	−0.14	0.040	166	7544 ± 10,123	−0.20	0.039	156	2580 ± 838	−0.23	0.016
Glucagon‐like peptide‐1												
Premeal (pmol/L)	263	31.0 ± 19.8	−0.26	<0.001	113	29.0 ± 25.7	−0.21	0.084	170	31.5 ± 13.5	−0.50	<0.001
Time‐to‐peak conc (min)	241	50.9 ± 24.1	−0.20	0.010	102	51.5 ± 24.0	−0.31	0.014	156	49.9 ± 24.2	−0.15	0.148
AUC (pmol·min/L)	263	2118 ± 1104	−0.17	0.019	113	2152 ± 1502	−0.22	0.063	170	2099 ± 677	−0.35	<0.001
Insulin												
Premeal (mU/L)	190	8.0 ± 10.8	−0.40	<0.001	40	3.5 ± 2.0	−0.40	0.076	170	8.5 ± 11.3	−0.44	<0.001
AUC (mU·min/L)	190	520 ± 600	−0.42	0.001	40	351 ± 173	−0.38	0.093	170	525 ± 632	−0.47	<0.001
GI/appetite perceptions (mm·min)												
AUC Hunger	390	−398 ± 1405	0.11	0.070	240	−682 ± 1545	0.14	0.076	170	51 ± 1046	0.02	0.855
AUC Desire to eat	390	−522 ± 1393	0.11	0.060	240	−857 ± 1538	0.18	0.019	170	28 ± 947	−0.04	0.702
AUC Prospective consumption	390	−597 ± 1302	0.16	0.009	240	−947 ± 1427	0.25	0.001	170	−63 ± 846	−0.02	0.835
AUC Fullness	390	1027 ± 1338	−0.10	0.090	240	1420 ± 1470	−0.11	0.164	170	437 ± 817	−0.08	0.381
AUC Nausea	390	224 ± 907	−0.23	<0.001	240	277 ± 1084	−0.31	<0.001	170	142 ± 495	−0.17	0.065
AUC Bloating	390	558 ± 1205	−0.27	<0.001	240	781 ± 1403	−0.37	<0.001	170	236 ± 727	−0.06	0.517

AUC, area under the curve; GI, gastrointestinal

a Variations in the number of study visits (*n*) for the various variables due to missing data

#### Models 1a and 1b (all studies)

Within the variables characterizing IPPWs, peak number, total number, and AUC of the number were strongly associated with each other (all *r* ≥ 0.83). Of these variables, AUC of the number was excluded from all multivariate models because it least characterized the IPPW response. Among the CCK variables, peak concentration and AUC were strongly correlated (*r* = 0.87), and peak concentration was included because it best described the hormone profile.

#### Models 2a and 2b (lipid studies)

Of the variables characterizing IPPWs in these studies, peak number, total number, and AUC of the number were strongly associated with each other (all *r* ≥ 0.82); thus, AUC of the number was excluded because it least characterized the IPPW response. For the BPP variables, peak pressure and AUC were strongly correlated (*r* = 0.85); thus, peak pressure was included because it showed a slightly stronger correlation with energy intake. Among the CCK variables, peak concentration and AUC were strongly correlated (*r* = 0.88), and peak concentration was included because it best characterized the CCK response. Within the GI/appetite perception variables, AUCs for hunger, desire to eat, and prospective consumption were correlated moderately with each other (all *r* ≤ 0.77); thus, AUC for prospective consumption was excluded.

#### Model 3 (protein studies)

All variables characterizing IPPWs were very strongly correlated (*r* ≥ 0.89); thus, total number was included in the multivariate model because it showed the highest F‐statistics. Of the BPP variables, peak pressure and AUC were strongly correlated (*r* = 0.70), and peak pressure was selected for inclusion because it showed a stronger correlation with energy intake. Within the CCK variables, peak concentration and AUC were strongly correlated (*r* = 0.91); thus, peak concentration was included because it best characterized the CCK response. AUCs for hunger, desire to eat, and prospective consumption were modestly associated with each other (all *r* ≤ 0.66); thus, hunger was included based on the highest F‐statistics. GLP‐1 and PYY premeal concentrations were strongly correlated with the corresponding AUCs (*r* ≥ 0.88); premeal concentrations were included because they showed stronger correlations with energy intake.

None of the other variables showed any multicollinearity, therefore, they were entered into the multivariable models automatically. For the protein and lipid‐based studies, additional variables were excluded on the basis of clinical utility and the smallest F‐statistics to reduce the models to a number of parameters appropriate for the reduced sample sizes.

### Multivariable mixed‐effects models

Model 1a (all variables measured across all studies plus PYY) identified antral amplitude (*P *<* *0.05), a trend for peak BPP (*P *=* *0.082), as well as bloating (*P *<* *0.05) as predictors of energy intake (Table 4); increases in antral amplitude or peak BPP by 1 mmHg, or in bloating by 1 mm·min, while controlling for all the other variables, were associated with an increase of 2.4 kJ, or decreases of ~37 kJ or ~0.2 kJ in energy intake, respectively. Model 1b (all variables measured across all studies plus GLP‐1) identified a trend for peak CCK concentration (*P *=* *0.074), premeal GLP‐1 concentration (*P *<* *0.01), and GLP‐1 AUC (*P *<* *0.01), and a trend for hunger (*P *=* *0.09) as determinants of energy intake. Increases in CCK or GLP‐1 by 1 pmol/L, in GLP‐1 AUC by 1 pmol·min/L or in hunger AUC by 1 mm·min were associated with reductions of ~66, ~27, and ~0.6 kJ, or an increase of ~0.3 kJ, in energy intake, respectively.

**Table 4:** Results of mixed‐effects multivariable models for determination of independent predictors of energy intake

Variable	All studies								Lipid studies								Protein studies			
	Model 1a (incl PYY)				Model 1b (incl GLP‐1)				Model 2a (incl PYY)				Model 2b (incl GLP‐1)				Model 3 (incl PYY & GLP‐1)			
	(*n* = 288)				(*n* = 263)				(*n* = 142)				(*n* = 82)				(*n* = 170)			
	b ± SEM	*β*	*t*	*P*	b ± SEM	*β*	*t*	*P*	b ± SEM	*β*	*t*	*P*	b ± SEM	*β*	*t*	*P*	b ± SEM	*β*	*t*	*P*
Antral pressure waves																				
Number, total	−1.4 ± 1.2	−0.06	−1.14	0.256	−0.4 ± 1.0	−0.03	−0.44	0.657	−1.7 ± 1.5	−0.09	−1.12	0.265	−	−	−	−	−2.7 ± 1.7	−0.11	−1.64	0.105
Amplitude, mean (mmHg)	2.4 ± 1.0	0.13	2.44	0.015	1.6 ± 1.2	0.09	1.38	0.170	–	–	–	–	–	–	–	–	1.8 ± 1.3	0.11	1.40	0.165
Isolated pyloric pressure waves																				
Number premeal/15 min	4.9 ± 7.2	0.04	0.68	0.494	−0.04 ± 12.3	0.00	0.00	0.997	−15 ± 8.0	0.13	1.92	0.057	–	–	–	–	−21 ± 15	−0.14	−1.37	0.175
Peak number/15 min	−12.2 ± 8.4	−0.11	−1.44	0.150	−9.9 ± 13.4	−0.08	−0.74	0.463	−15.2 ± 9.3	−0.14	−1.63	0.105	−8.1 ± 19.3	−0.07	−0.42	0.675	–	–	–	–
Time‐to‐peak number (min)	–	–	–	–	–	–	–	–	–	–	–	–	–			–	–	–	–	–
Total number	0.04 ± 2.4	0.00	0.02	0.987	0.2 ± 4.3	0.01	0.06	0.956	1.2 ± 2.3	0.06	0.53	0.598	1.9 ± 4.3	0.07	0.44	0.664	2.2 ± 4.5	0.05	0.48	0.629
AUC number (min)	–		–	–	–	–	–	–	–	–	–	–	–	–	–	–	–	–	–	–
AUC amplitude (mmHg·min)	−0.02 ± 0.07	−0.02	−0.32	0.752	−0.01 ± 0.09	−0.01	−0.12	0.906	–	–	–	–	–	–	–	–	–	–	–	–
Basal pyloric pressures																				
Peak pressure (mmHg)	−37 ± 21	−0.11	−1.75	0.082	−28 ± 29	−0.07	−0.95	0.346	−32 ± 17.0	−0.10	−1.85	0.067	69 ± 35	0.17	1.96	0.055	−48 ± 24	−0.13	−2.01	0.047
Time‐to‐peak pressure (min)	–	–	–	–	–	–	–	–	–	–	–	–	–	–	–	–	–	–	–	–
AUC (mmHg·min)	0.28 ± 0.51	0.03	0.55	0.584	0.77 ± 0.71	0.08	1.09	0.277	–	–	–	–	–	–	–	–	–	–	–	–
Duodenal pressure waves																				
Number, total	−0.07 ± 0.29	−0.01	−0.25	0.806	0.05 ± 0.3	0.01	0.14	0.885	0.1 ± 0.34	0.02	0.29	0.770	0.3 ± 0.3	0.08	0.99	0.329	–	–	–	–
Amplitude, mean (mmHg)	−1.7 ± 2.5	−0.05	−0.68	0.503	−0.3 ± 2.8	−0.01	−0.11	0.911	5.4 ± 3.0	0.16	1.81	0.072	–	–	–	–	–	–	–	–
Ghrelin^a^																				
Premeal (pmol/L)	–	–	–	–	–	–	–	–	–	–	–	–	–	–	–	–	0.8 ± 0.8	0.10	0.90	0.382
AUC (pmol·min/L)	–	–	–	–	–	–	–	–	–	–	–	–	–	–	–	–	–	–	–	–
Cholecystokinin																				
Premeal (pmol/L)	–	–	–	–	–	–	–	–	–	–	–	–	–	–	–	–	–	–	–	–
Peak concentration (pmol/L)	−1.8 ± 19.2	−0.01	−0.09	0.927	−66 ± 37	−0.13	−1.80	0.074	−3 ± 18.0	−0.01	−0.18	0.860	−80 ± 43	−0.20	−1.87	0.066	11 ± 68	0.02	0.17	0.869
Time‐to‐peak conc (min)	–	–	–	–	–	–	–	–	–	–	–	–	–	–	–	–	–	–	–	–
AUC (pmol·min/L)	–	–	–	–	–	–	–	–	–	–	–	–	–	–	–	–	–	–	–	–
Peptide YY																				
Premeal (pmol/L)	−3.0 ± 3.7	−0.05	−0.81	0.417	–	–	–	–	−3.2 ± 4.1	−0.07	−0.78	0.436	–	–	–	–	−2.2 ± 9.4	−0.02	−0.23	0.819
AUC (pmol·min/L)	0.02 ± 0.02	0.09	0.98	0.330	–	–	–	–	0.01 ± 0.02	0.09	0.68	0.498	–	–	–	–	–	–	–	‐
Glucagon‐like peptide‐1																				
Premeal (pmol/L)	–	–	–	–	−27.1 ± 9.9	−0.33	−2.75	0.007	–	–	–	–	4.4 ± 5.8	0.07	0.76	0.451	–8.7 ± 13.6	−0.07	−0.64	0.525
Time‐to‐peak conc (min)	–	–	–	–	−2.0 ± 3.4	−0.03	−0.58	0.560	–	–	–	–	−11 ± 4.0	−0.16	−2.46	0.018	4.9 ± 4.4	0.07	1.12	0.268
AUC (pmol·min/L)	–	–	–	–	0.57 ± 0.19	0.38	2.97	0.003	–	–	–	–	–	–	–	–	–	–	–	–
Insulin																				
Premeal (mU/L)	–	–	–	–	–	–	–	–	–	–	–	–	–	–	–	–	−17 ± 11	−0.13	−1.55	0.125
AUC (mU·min/L)	–	–	–	–	–	–	–	–	–	–	–	–	–	–	–	–	–	–	–	–
GI/appetite perceptions (mm·min)																				
AUC Hunger	0.01 ± 0.08	0.01	0.15	0.881	0.25 ± 0.15	0.19	1.68	0.094	−0.01 ± 0.08	−0.01	−0.13	0.894	0.4 ± 0.2	0.41	1.91	0.061	0.1 ± 0.1	0.06	0.89	0.378
AUC Desire to eat	−0.07 ± 0.11	−0.07	−0.70	0.483	−0.11 ± 0.16	−0.09	−0.68	0.499	−0.04 ± 0.09	−0.04	−0.43	0.670	−0.3 ± 0.2	−0.29	−1.30	0.198	–	–	–	–
AUC Prospective consumption	0.09 ± 0.11	0.08	0.87	0.385	0.004 ± 0.15	0.00	0.03	0.978	–	–	–	–	–	–	–	–	–	–	–	–
AUC Fullness	−0.03 ± 0.07	−0.03	−0.51	0.612	−0.07 ± 0.11	−0.05	−0.62	0.537	–	–	–	–	–	–	–	–	–	–	–	–
AUC Nausea	−0.11 ± 0.08	−0.07	−1.39	0.166	−0.19 ± 0.12	−0.12	−1.63	0.105	−0.12 ± 0.07	−0.10	−1.56	0.122	−0.2 ± 0.1	−0.20	−1.71	0.094	−0.1 ± 0.2	−0.04	−0.64	0.522
AUC Bloating	−0.17 ± 0.07	−0.14	−2.49	0.014	0.03 ± 0.13	0.02	0.24	0.811	−0.23 ± 0.06	−0.25	−3.67	0.000	−0.1 ± 0.2	−0.13	−0.81	0.421	−0.1 ± 0.2	−0.03	−0.31	0.756

n, number of visits; AUC, area under the curve; GI, gastrointestinal; GLP‐1, glucagon‐like peptide‐1; PYY, peptide Y.

a Only included in “Protein studies” model due to insufficient data from lipid studies.

Model 2a (lipid‐based studies including PYY) identified trends for number of premeal IPPWs (*P *=* *0.057), peak BPP (*P *=* *0.067), and duodenal amplitude (*P *=* *0.072), as well as bloating (*P *<* *0.001), as predictors of energy intake, so that increases by *n* = 1, 1 mmHg, or 1 mm·min were associated with decreases of 15 kJ or 32 kJ, an increase of 5.4 kJ, or a decrease of ~0.2 kJ, in energy intake, respectively (Table 4). In model 2b (lipid‐based studies including GLP‐1), increases in peak BPP and plasma CCK peak concentration were associated with trends for an increase (*P *=* *0.055), or a decrease (*P *=* *0.066), in energy intake, respectively; an increase in peak BPP by 1 mmHg was associated with an increase in energy intake of ~69 kJ, whereas an increase in plasma CCK peak concentration by 1 pmol/L reduced energy intake by ~80 kJ. The time‐to‐peak concentration of plasma GLP‐1 was also associated with energy intake (*P *<* *0.05); an increase by 1 min reduced energy intake by ~11 kJ. Finally, there were trends for hunger (*P *=* *0.061) and nausea (*P *=* *0.094) to be associated with energy intake; increases in the hunger or nausea AUC by 1 mm·min were associated with an increase, or decrease, in energy intake of 0.4 kJ, or 0.2 kJ, respectively.

Model 3 (all variables measured in the protein‐based studies) only identified peak BPP as an independent determinant of energy intake (*P *<* *0.05; Table 4); thus, an increase by 1 mmHg was associated with a decrease in energy intake of ~48 kJ.

## Discussion

This study has evaluated, using a pooled‐data analysis, the GI motor and hormone determinants of acute energy intake in response to intraduodenal administration of nutrients, particularly protein and lipid. The major findings are that the models, based either on all studies or the lipid studies, identified a range of GI factors, including amplitude of antral pressure waves, premeal GLP‐1 and time‐to‐peak GLP‐1 concentrations, GLP‐1 AUC and bloating scores, and trends for BPP, amplitude of duodenal pressure waves, peak CCK concentrations, and hunger and nausea scores, as independent determinants of energy intake. In contrast, in the model that included the protein studies only BPP was identified as an independent determinant of energy intake. Moreover, the effects of variables identified as independent determinants were small. That said, it is important to recognize that the reported effects relate to a change of 1 unit for each variable, for example, an increase in plasma CCK by 1 pmol/L was associated with a reduction in energy intake of ~66–80 kJ across models, and lipid‐stimulated plasma CCK may increase by 3–5 pmol/L. Taken together, our observations indicate that (i) the contributions of GI factors to energy intake regulation are nutrient specific and (ii) while GI mechanisms are important in the regulation of acute energy intake (Cummings and Overduin 2007; Steinert et al. 2013), this effect most likely reflects the outcome of relatively small, cumulative contributions from a range of interrelated factors. A clear implication is that ‘GI’ strategies for the management of obesity are unlikely to prove effective if only one mechanism is targeted.

The studies included in this analysis utilized intraduodenal nutrient infusions, which allow the delivery of nutrients into the proximal small intestine (the major location of nutrient sensors, from which signals to initiate feedback control of upper GI motility, slowing of gastric emptying, and gut hormone release, are initiated) to be standardized. The rationale for this approach is that it excludes the confounding effects of the substantial interindividual variation in gastric emptying and ‘bypasses’ gastric distension which modulates energy intake. In addition, the delivery of nutrients prior to a meal “mimics” the preload concept, which aims to administer nutrients that stimulate specific GI functions to influence outcomes (e.g., energy intake or blood glucose) in response to that meal (Gentilcore et al. 2006). We infused long‐chain triglyceride emulsions, whey protein, or their digestive products, fatty acids, and amino acids, as these have consistently been shown, in studies from our laboratory and those of others, to suppress subsequent energy intake in humans when given acutely (Matzinger et al. 2000; Feltrin et al. 2004; Ryan et al. 2013).

Our main hypothesis was that with protein, unlike lipid, GI factors were not the major driving force in determining subsequent acute energy intake. Indeed, our analysis identified a range of factors as contributors to energy intake regulation in response to intraduodenal lipid, whereas only a single factor, BPP, was identified for protein, suggesting that other factors are more important in the case of protein. Although these could not be determined in this study, important roles for, for example, diet‐induced thermogenesis (Westerterp‐Plantenga et al. 1999), intestinal gluconeogenesis (Mithieux 2009), and direct effects of elevated plasma amino acid concentrations within the brain (Tome 2004), have been identified by others.

The increased recognition of the fundamental role for the upper GI tract in regulating energy intake, but also blood glucose, has prompted extensive research into potential therapeutic agents that target these mechanisms. Among the gut peptides, CCK, PYY, and GLP‐1 have received much interest, due to their satiating effects when infused intravenously in healthy or obese humans, however, as has been shown in the case of PYY(3‐36), the satiating effects could only be achieved at doses that elevated plasma PYY concentrations to supraphysiological levels and also induced nausea (Degen et al. 2005). Investigations of the role of endogenous gut hormones have been more limited, due to the relative lack of specific receptor antagonists for use in humans. Studies using the CCK‐A receptor antagonist, loxiglumide, have shown that blockade of CCK‐A receptors has only a modest (Beglinger et al. 2001), or no (Lieverse et al. 1995), effect to increase energy intake, suggesting that while endogenous CCK may play a role, its individual contribution is small. GLP‐1 agonists that stimulate insulin in a glucose‐dependent manner, but also slow gastric emptying, and, thus, small intestinal glucose delivery and absorption, are now widely used in the management of type 2 diabetes (Nauck et al. 1997). Their use is also associated with a modest reduction in body weight in obese patients with and without type 2 diabetes, and the GLP‐1 agonist, liraglutide, in a dose of 3.0 mg, has recently been approved for management of obesity in humans. However, these drugs are expensive and associated with adverse effects, particularly nausea and diarrhea (Horowitz et al. 2008), and their longer‐term safety remains uncertain. Their limited effect to decrease body weight may reflect the targeting of only one mechanism.

The notion that there is not a “dominant” GI factor, but that numerous factors interact, to suppress energy intake, is not surprising, considering that under physiological conditions a range of hormones are released, and other functions, including motility, are stimulated. For example, we have shown that the potent suppressive effects of specific nutrients, when administered intraduodenally, particularly the fatty acid, lauric acid, and also the amino acid, l‐tryptophan, on energy intake are associated with marked effects on the release of a number of gut hormones and modulation of upper gut motility (Feltrin et al. 2004; Steinert et al. 2014). Further evidence for the role of interrelated GI functions in the regulation of energy intake comes from our previous pooled data analysis (Seimon et al. 2010), in which the magnitude of stimulation of both pyloric pressures and plasma CCK was identified as major independent determinants of subsequent energy intake. In contrast to this previous analysis, we were, however, unable to identify any specific parameter as a major determinant of subsequent energy intake. Instead, our data suggest that a number of factors make small contributions to determine energy intake. There was also an inconsistency; in model 2b (lipid‐based studies that measured GLP‐1), an increase in peak BPP by 1 mmHg was associated with a small increase, rather than a decrease, in energy intake, which is difficult to reconcile with our other findings. However, the statistical analysis used considers each factor while keeping all other variables constant, occasionally resulting in nonintuitive outcomes.

Our observations are clinically relevant, given that they provide evidence as to why any treatments that only target one pathway, for example, administration of a particular gut hormone, or its analog, are not, in the absence of aversive effects, very effective in reducing energy intake and, in the longer‐term, body weight. A number of studies have investigated combination approaches (Gutzwiller et al. 2004; Neary et al. 2005; Steinert et al. 2010) and found that oral or intravenous administration of combinations of gut hormones suppressed hunger or energy intake more than individual gut hormones (reviewed in (Steinert et al. 2016)). For example, in a recent study, combined intravenous infusion of GLP‐1 and PYY(3‐36) reduced energy intake compared with placebo more than the sum of infusion of either hormone alone (Schmidt et al. 2014). A similar approach has been used for combinations of centrally acting drugs, for example, phentermine and topiramate, although their side‐effects are considerable (Bray et al. 2016).

Our study has a number of limitations, which should be taken into account in interpreting our data. Some gut hormones that may modulate eating were either not measured (e.g., glucagon (Geary et al. 1992), pancreatic polypeptide (Jesudason et al. 2007)), or measured only in a small number of studies (e.g., ghrelin, insulin), and it remains uncertain how their inclusion in the analyses may have affected outcomes. These and other GI, or extra‐GI, mechanisms, not taken into account in our studies, including plasma amino acid concentrations, warrant further investigation in prospective studies. As in our previous study (Seimon et al. 2010), PYY was not identified as an independent determinant of energy intake, but PYY was only assessed in a subset of studies, hence, the analyses may have not been sufficiently powered. That said, GLP‐1 data were also available only in some studies, yet, GLP‐1 concentrations were identified as an independent determinant of subsequent energy intake. The studies were performed in healthy, young men, hence, we cannot be certain that the findings can be extended to women, overweight, obese, or elderly individuals, although this is intuitively unlikely. As gastric distension is involved in the acute regulation of energy intake, and enhances the effect of intraduodenal nutrients on GI perceptions (Feinle et al. 1997), the magnitude of effects identified in our analyses may be greater in the presence of gastric distension. Some selection of parameters to be included into the models was required. Therefore, it is possible that significant effects may have been confounded with other parameters of the same outcome (e.g., for peak vs. AUC vs. mean data of a variable). A statistical comparison of the lipid and protein cohorts to formally test for a difference in the independent determinants was not possible due to the available sample size. The comparison of results between these two subgroups is therefore qualitative only. Finally, principle components analysis, as has been used recently in a study to evaluate gastrointestinal and psychological traits associated with obesity (Acosta et al. 2015), was not deemed an appropriate analysis method in the case of our data, as our data were collected over multiple visits in each subject and there was clustering of subjects within separate studies.

In conclusion, our analysis indicates that a number of GI factors, including GI pressures, plasma CCK, and GLP‐1 concentrations and some GI/appetite perception variables, are determinants of energy intake at a subsequent meal in response to intraduodenal lipid, but to a much lesser extent, protein. However, individual effects were small, therefore, the overall effect on energy intake, particularly by lipid, most likely reflects small, cumulative contributions from a combination of these, interrelated, factors. The findings have implications for the design of effective approaches for the management of obesity that target the GI tract, that is, these are unlikely to be effective if only one mechanism is targeted.

## Conflict of Interest

None of the authors have any conflicts of interests to declare.
